# Photobiomodulation mechanisms: duration of action in the human prefrontal cortex

**DOI:** 10.3389/fnbeh.2025.1726805

**Published:** 2025-12-11

**Authors:** Patrick O’Connor, Turner Lime, Douglas W. Barrett, F. Gonzalez-Lima

**Affiliations:** 1 Department of Psychology, Texas Consortium in Behavioral Neuroscience, The University of Texas at Austin, Austin, TX, United States; 2 Department of Psychiatry and Behavioral Sciences, Dell Medical School, The University of Texas at Austin, Austin, TX, United States

**Keywords:** prefrontal cortex, photobiomodulation, transcranial infrared laser stimulation, functional near-infrared spectroscopy, functional connectivity, PBM

## Abstract

**Introduction:**

Transcranial infrared laser stimulation (TILS) is a form of photobiomodulation (PBM) using a wavelength of 1064 nm shown to enhance metabolic and hemodynamic activity in the human prefrontal cortex (PFC). Prior studies have shown that when applied to the PFC in the right hemisphere, TILS improves PFC-based memory and learning and sustains attention and mood in healthy adults. However, the temporal duration of PBM mechanisms following a single administration remains poorly understood in humans. The objective of this study was to evaluate the duration of functional connectivity effects of a single administration of TILS to the right anterior PFC during both resting-state and memory-activated conditions over a 5-day period.

**Methods:**

Hemodynamics-derived functional connectivity of the PFC in 12 healthy adults was measured using a 48-channel functional near-infrared spectroscopy (fNIRS) during 5-min resting-state and 2-back memory task activation phases, collected at six time points over a 5-day span. A sham-controlled, within-subject crossover design was employed: all participants received both sham and active TILS in counterbalanced order, with a 4-week washout period between sessions.

**Results:**

Relative to sham, a single administration of TILS significantly modulated PFC functional connectivity during cognitively demanding memory tasks across the 5-day assessment period. No significant effects were observed during resting-state measurements. No adverse effects were reported.

**Discussion:**

These findings suggest that a single administration of TILS can induce functional neuroplasticity in the PFC that persists for several days. The results advance understanding of PBM mechanisms and may inform future interventions aimed at promoting longer-lasting neurocognitive benefits.

## Introduction

Interventions purported to augment cognitive functioning are of growing scientific interest and hold enormous potential for research and promotion of cognition and brain wellness, neurological health, and psychological outcomes (Dresler et al., 2019). Despite side effects and contraindications, the most widely studied putative neurocognitive enhancers are biochemical and pharmaceutical (Sarter, 2006; Hofmann et al., 2011; Tricco et al., 2013; Dresler et al., 2019). In this respect, transcranial infrared laser stimulation (TILS), a form of photobiomodulation (PBM), is of particular interest and promise, as it induces a variety of positive neurocognitive and psychological effects without side effects or contraindications (Barrett and Gonzalez-Lima, 2013; Rojas and Gonzalez-Lima, 2013; Gonzalez-Lima and Barrett, 2014; Gonzalez-Lima, 2021; Wang et al., 2024).

Transcranial infrared laser stimulation is characterized by the application of directional low-power and high-fluence monochromatic 1064 nm infrared light to the forehead. A fraction of this light penetrates superficial tissue and the skull before it is ultimately absorbed by mitochondria-rich cortical tissue (Tedford et al., 2015; Tian et al., 2020; Huang et al., 2021), resulting in increased metabolism and hemodynamics of the prefrontal cortex (PFC) (Tian et al., 2016; Wang et al., 2017; Saucedo et al., 2021).

Transcranial infrared laser stimulation has been found to augment memory, attention, learning, and executive function (Rojas et al., 2012; Barrett and Gonzalez-Lima, 2013; Disner et al., 2016; Blanco et al., 2017a,b; Holmes et al., 2019; Zhao et al., 2022). There is also limited evidence that TILS may improve mood (Schiffer et al., 2009; Barrett and Gonzalez-Lima, 2013; Disner et al., 2016). Despite these promising findings, TILS remains understudied and underutilized in part due to uncertainty with respect to the duration of its physiological effects in the human brain *in vivo*.

Recent TILS studies utilizing near-infrared spectroscopy (NIRS) have demonstrated that a single administration of TILS results in local increases in concentrations of oxidized cytochrome-c-oxidase (CCO), the rate-limiting enzyme in the mitochondrial electron transport chain, as well as oxygenated hemoglobin (HbO) during and immediately following stimulation (Tian et al., 2016; Wang et al., 2017; Holmes et al., 2019; Pruitt et al., 2020; Saucedo et al., 2021). Further, these effects are not relegated to the site of stimulation; contralateral effects have also been observed (Tian et al., 2016; Holmes et al., 2019; Saucedo et al., 2021). Local excitation of cortical tissue may bring about a metabolic response in other brain regions via excitatory neurotransmission and CCO induction (Gonzalez-Lima and Cada, 1998; Wong-Riley et al., 1998). The duration of metabolic and hemodynamic effects of TILS in humans has not yet been investigated in large part due to an inability of NIRS to obtain absolute concentration values of CCO and HbO, making across-session comparisons difficult to assess in humans. However, long-lasting neuroplastic CCO changes from a single TILS administration have been found in the rat brain (Wade et al., 2023).

Functional near-infrared spectroscopy (fNIRS) is an application of NIRS that allows for analysis of functional connectivity via the temporal synchronization of hemodynamic HbO changes over time (Villringer and Dirnagl, 1995; Lu et al., 2010). Measures of functional connectivity including Pearson correlation coefficients allow for comparisons both between subjects and across discrete measurement acquisition periods. The aim of this study was to investigate the effects of a single session of TILS on functional connectivity of the PFC (as measured by fNIRS) across 5 days. We hypothesized that TILS would result in greater desynchrony (lower Pearson correlation coefficients) relative to sham, that these effects would be greater during a cognitive activation task relative to resting state, and that these effects would be decreasing with time thereafter. Resting states are characterized by synchrony of brain oscillations, while activation states have more desynchronized activity (Lee and Dan, 2012). Assessing brain activity during both rest and during a cognitive activation task (the 2-back task) allows for a comparison between the effects of TILS on functional connectivity between these two brain states, with the cognitive task driving the use of PFC metabolic resources greater than that used at rest. However, performance on the 2-back memory task is not hypothesized to change as a result of TILS, as performance is expected to quickly reach ceiling in healthy adults.

## Methods and methods

### Participants

12 healthy adult participants (*N* = 5 female, 7 male; *M* = 29.5 years old, SD = 7.7) were recruited for this study. All procedures were reviewed and approved by the University of Texas at Austin (UT) Institutional Review Board and complied with NIH guidelines on human research. Participants who met the following inclusionary criteria were enrolled in the study: (1) 18 years of age or older, (2) not currently pregnant, (3) no recent history of TILS (5 weeks). Written informed consent was obtained from each participant before beginning the experiment.

### Study design and apparatus

**FIGURE 1:**
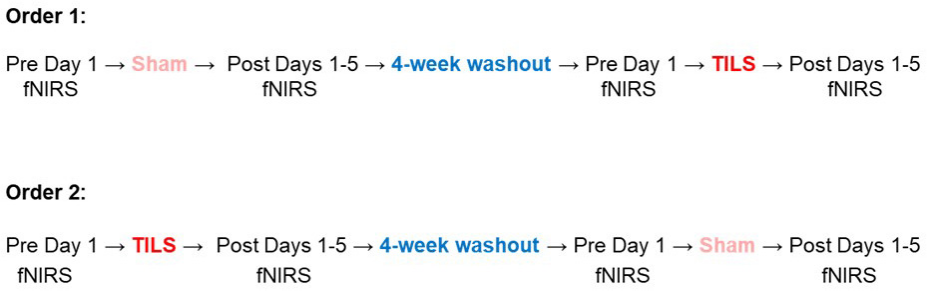
Experimental time course for participants randomized to one of two orders of procedure. Neuroimaging with fNIRS was conducted pre- and post-TILS on day 1 and for four consecutive days following. At each neuroimaging timepoint, participants engaged in a 5-min rest and 5-min cognitive activation task. A randomized crossover design was utilized such that all 12 participants underwent both active TILS and sham with a 4-week washout period.

Using a single-blind, sham-controlled design, consenting participants were stratified by sex and then randomly assigned to either TILS or sham to the right anterior PFC. A crossover design was implemented such that participants repeated the experiment in the opposite condition after a 4-week washout period. Figure 1 depicts the experimental time course for each order of procedure.

Except for sequencing of sham and active TILS (procedure order), all experimental procedures were identical between subjects. All researchers completed laser safety training provided by the University of Texas at Austin’s Environmental Health and Safety office, which also approved the standard operating procedure for the laser. Both participants and researchers wore protective eyewear during TILS/sham administration. Participants were also instructed to close their eyes during TILS/sham administration.

**FIGURE 2:**
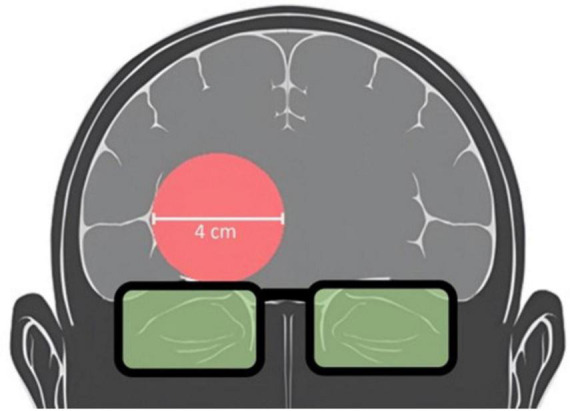
Schematic of laser placement targeting the anterior PFC. Participants received TILS centered on the right forehead above the eyebrows. TILS light aperture (red circle) and protective goggles over eyes (green) are shown.

The PBM stimulation (TILS) and hemodynamic neuroimaging (fNIRS) devices were each optically based. Interference and occlusion were prevented by utilizing the two devices at separate, non-overlapping time intervals. TILS used a well-collimated laser diode emitting light at a wavelength of 1064 nm (CytonPro-5000, CytonSys Inc., Austin, Texas, USA). The monochromatic, continuous-wave light was emitted in a 4-cm diameter circular flat-top beam (Amaroli et al., 2021) with an area of 13.6 cm^2^. The power output was 3.4 watts, and the irradiance (power density) was 250 mW/cm^2^. Active laser exposure totaled 480 s (8 min of continuous application) directed at a right forehead site centered at the frontal polar EEG electrode placement (Fp2) over the anterior PFC (Brodmann area 10). Fluence dose (energy density) was 120 J/cm^2^ and energy was 1,632 J. A red guiding light at 650 nm (0.08 mW/cm^2^) provided a visual aid for the targeting of the stimulation site and remained on for all 8 min of TILS/sham administration. The laser aperture was fixed in place 5 centimeters from the participants forehead above the right eyebrows. The sham consisted of the 650 nm targeting light without TILS at 1064 nm. Figure 2 schematically illustrates the area of TILS/sham used for each subject.

**FIGURE 3:**
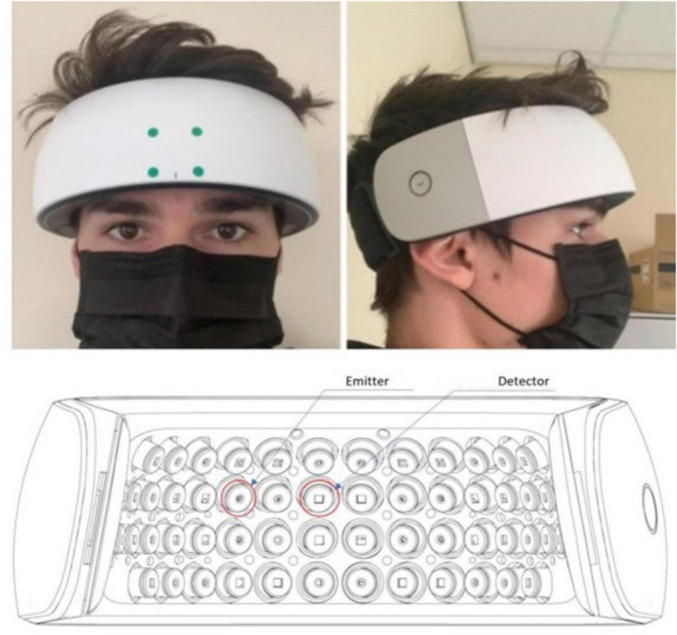
Top panels show photos of a model wearing the fNIRS device used to measure hemodynamics in the PFC. Bottom panel depicts schematic of the emitters (circle points) and detectors (square points) on the surface of the device contacting the participant’s forehead. The device provided 48 measurement channels with a 3 cm separation between emitters and detectors. Participants were instructed to sit in a relaxed and upright position and to refrain from making rapid movements or talking during neuroimaging to minimize motion artifacts. fNIRS neuroimaging was performed independently to TILS because it is not physically possible to use the two procedures on the forehead at the same time.

Changes in cerebral hemodynamics were measured non-invasively via a comfortable wearable device designed for functional near-infrared spectroscopy (fNIRS) of the PFC (NIRSIT, OBELAB Inc., Seoul, S. Korea). The device provided 24 dual-wavelength emitters of light (780 nm and 850 nm wavelengths, not exceeding 1 mW in power) as well as 32 photon detectors. 48 channels with an emitter-detector separation of 3 cm were utilized in our analysis. The wearable fNIRS device was centrally placed on the participant’s forehead and gently secured via an elastic strap around the participant’s head. Care was taken to prevent occlusion by hair. The device was connected via USB to a nearby laptop, running software (NIRSIT PC, OBELAB Inc.) which provided for device calibration and data collection. This software synchronized with the software E-Prime, which was used for the presentation of stimuli and timestamping of fNIRS neuroimaging data (task start and end). Figure 3 depicts the fNIRS device and placement on the forehead.

### Experimental protocol

Before undergoing TILS/sham, participants sat comfortably in a supportive chair while researchers affixed the fNIRS device. After the device was secured on the forehead and calibrated, participants next underwent non-invasive fNIRS neuroimaging during a 5-min rest phase and a 5-min cognitive activation task. Participants were instructed to remain reasonably still and to fix their gaze at the screen of a laptop directly in front of them while researchers waited out of sight to minimize distraction. The rest phase was characterized by the presentation of a fixation cross on an otherwise blank screen. During this phase, participants were instructed to relax and maintain their gaze on the screen.

**FIGURE 4:**
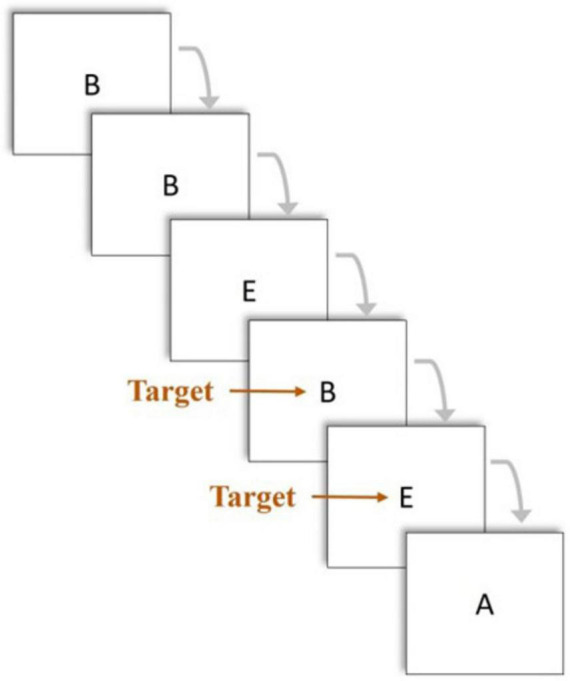
The 2-back memory task was used for the PFC cognitive activation phase. Participants were presented with one letter at a time and instructed to click the trackpad of the laptop only if the letter displayed on the screen in the current trial (n) matched the letter presented two trials earlier (n-2).

A cognitive activation phase in the form of a 2-back memory task was presented immediately following the rest phase. This task is a widely used measure for the assessment of working memory function (Owen et al., 2005; Kane et al., 2007). Participants were presented with one letter at a time and instructed to click the laptop’s trackpad when the letter presented on the current trial (n) matched the letter presented two trials previously (n-2). Researchers checked for participant comprehension of the task and instructions before beginning fNIRS setup. Neuroimaging with fNIRS during the rest and cognitive activation phases was repeated immediately following sham or TILS on Day 1 and once daily for four additional consecutive days (Days 2–5). Figure 4 depicts the 2-back cognitive activation task.

### Data processing and statistical analysis

For the 2-back activation task, hits percentage (correct responses to target trials divided by total target trials) was extracted from the behavioral data to allow for validation of participant engagement and stable cognitive performance during fNIRS measurements. Inspection of the behavioral data and outlier analysis resulted in the identification of one individual whose performance met outlier criteria (hits percentage greater than three standard deviations from the mean) on two sessions which may have been indicative of being distracted and/or poorly engaged in the task. As a result, we decided to eliminate this subject from functional connectivity analysis specific to the activation task (but not that specific to the rest). Repeated measures ANCOVA was conducted with participant sex and procedure order as between-subject factors and mean-centered age as a covariate to evaluate 2-back performance before and after sham or TILS. Results were regarded as statistically significant when *p* < 0.05 two-tailed.

Processing of the raw fNIRS data (detected light intensity) began with rejection of channels with a measured signal-to-noise ratio (SNR) below 30. Spectral data were then subjected to a low-pass filter of 0.1 Hz to mitigate noise from respiration and heart rate and a high-pass filter of 0.005 Hz to mitigate noise that may occur from device measurement drift and/or other phenomena resulting in slow changes unrelated to our signal of interest. Raw spectral data were next transformed into changes in concentration of oxygenated hemoglobin using the modified Beer-Lambert law (Delpy et al., 1988). Next, we manually removed channels that were visually aberrant consistent with a large amount of noise (outlier removal). Rejected channels were replaced with measurements of redundant channels sampling the same location when available. Otherwise, rejected channels were interpolated with the mean from nearby channels. We next generated 48 × 48 correlation matrices from the oxygenated hemoglobin signal for each participant at each time point. Separate matrices were generated for rest and cognitive activation task blocks. To avoid multiple comparisons in our statistical analysis, we performed data reduction by first transforming these values to z-scores (Fisher z-transformation) (Silver and Dunlap, 1987) and averaging all 1128 channel-pairs before transforming back to an all-channels averaged Pearson correlation coefficient (r), which served as an index of overall functional connectivity across the PFC. This approach was used in our recent study showing long-term changes in functional connectivity as a result of repeated TILS in a population of remitted bipolar participants (Barrett et al., 2025). One subject was missing data at a single timepoint due to measurement error. This value was interpolated using the mean of the participant’s r scores from their two adjacent data collection sessions. Statistical analysis of prefrontal average r scores was conducted for rest and cognitive task phases separately. Repeated measures ANCOVA with participant sex and procedure order as between-subject factors and mean-centered age as a covariate was utilized to evaluate TILS effects on functional connectivity. Results were regarded as statistically significant when *p* < 0.05 two-tailed. Effect size for repeated measures analyses were expressed as partial eta squared (η^2^_p_).

## Results

**FIGURE 5:**
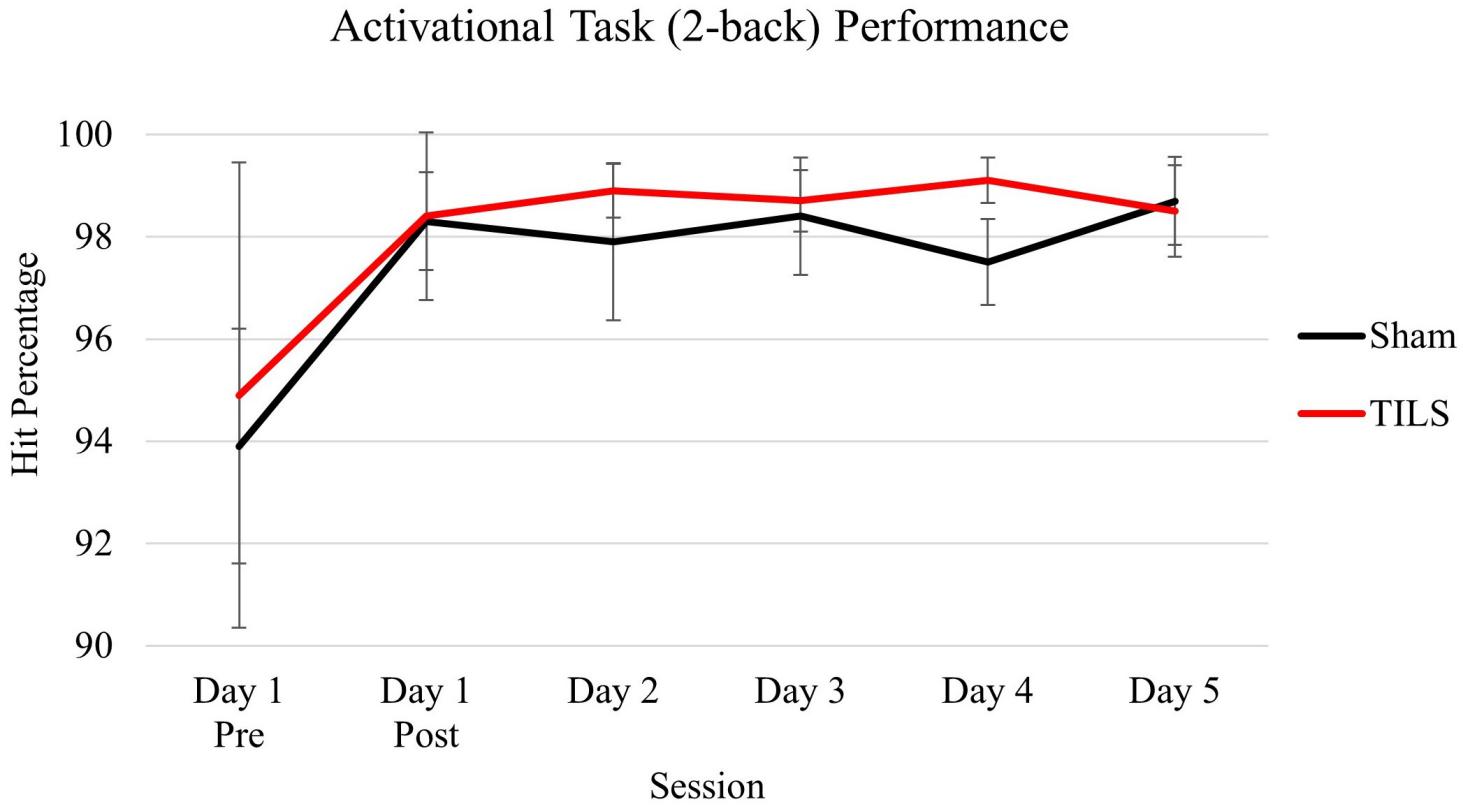
Means of cognitive activation task (2-back) performance as measured by hit percentage. Vertical bars indicate standard error.

As expected, participants reached ceiling behavioral performance after one administration of the 2-back memory task and maintained their stable behavioral performance thereafter. Consistent with our hypothesis, no significant differences were found on 2-back performance (hit percentage) between sham and TILS conditions across the 5 days. A main effect of session was found [*F*(5,30) = 2.95, *p* = 0.028] which was driven by an increase in hit percentage between the first and second administrations of the task corresponding to pre-TILS/sham and post-TILS/sham on Day 1, respectively. Figure 5 depicts means and standard errors of 2-back hit percentage.

Omnibus testing of the rest phase functional connectivity via repeated measures ANCOVA across all time points revealed no significant effects of TILS. Consequently, no further analysis of rest phase data was conducted.

In contrast, omnibus testing of the cognitive activation task phase functional connectivity across all timepoints indicated a significant TILS/sham by session interaction [*F*(5,30) = 2.88, *p* = 0.031, η^2^_p_ = 0.324], with mean correlations changing in the hypothesized direction. Consequently, repeated measures ANCOVA were repeated to evaluate an acute effect of TILS (Day 1 pre to post) as well as a prolonged effect of TILS (Day 1 Pre and Days 2–5).

Repeated measures ANCOVA with timepoints Day 1 Pre and Day 1 Post revealed a significant three-way interaction of TILS/sham, session, and procedure order [*F*(1,6) = 7.15, *p* = 0.037, η^2^_p_ = 0.544]. However, the interaction of TILS/sham by session was not significant [*F*(1,6), = 3.09, *p* = 0.129], indicating that our study did not detect a significant acute effect of TILS on Day 1.

Statistical analysis of the prolonged effect of TILS (Days 1–5, omitting Day 1 post) revealed a significant session by TILS/sham interaction [*F*(4,24) = 3.03, *p* = 0.037, η^2^_p_ = 0.336], indicating that our study detected a significant progressive effect of TILS on Days 1–5.

**FIGURE 6:**
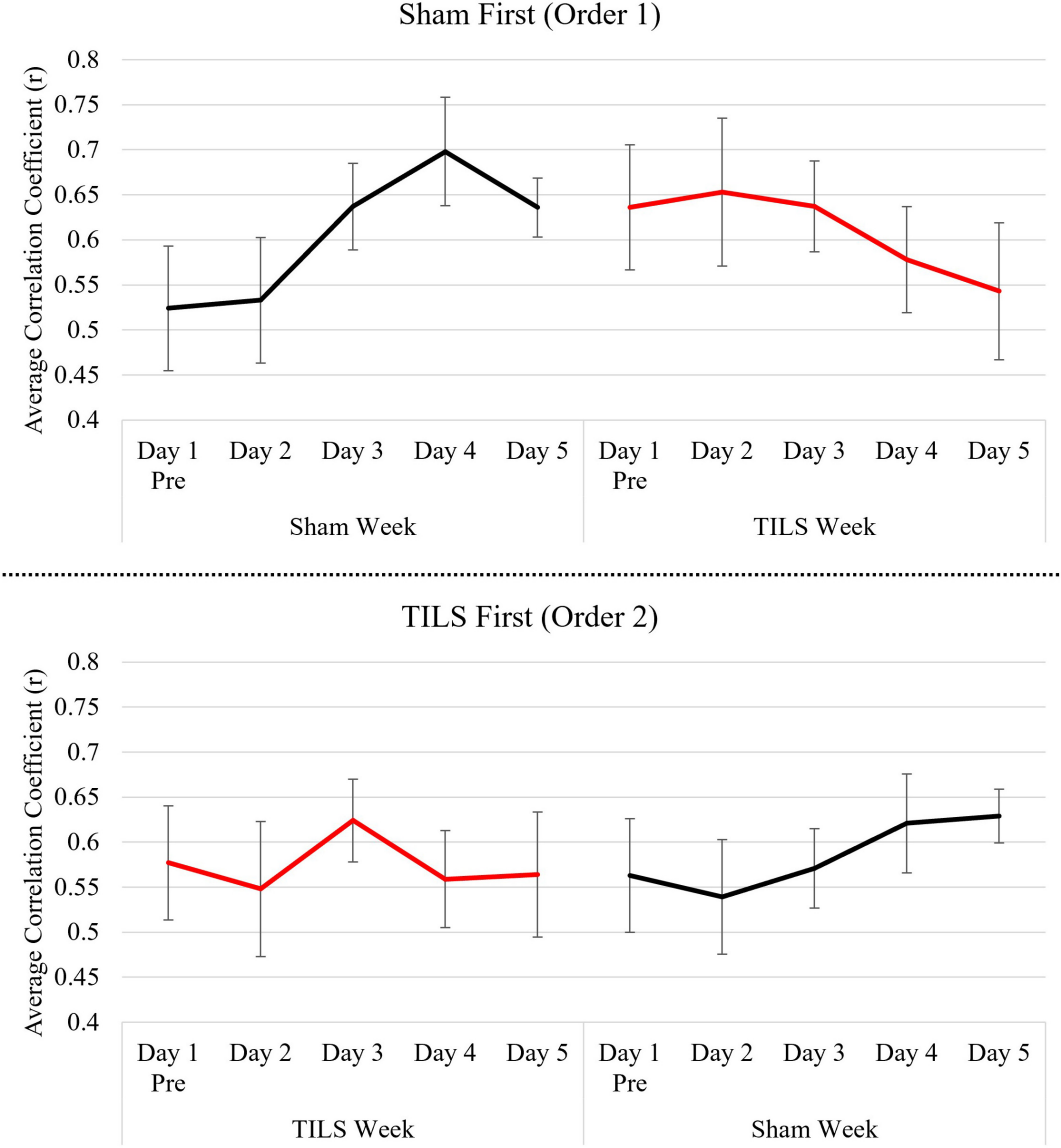
Means of PFC average correlation coefficient (r) across 5 days split by procedure order. Vertical bars indicate standard error.

Figures 6 depicts estimated marginal means of PFC average correlation coefficient (r) across 5 days, split by procedure order (sham-then-TILS or TILS-then-sham).

## Discussion

In the context of this fNIRS study, functional connectivity measured by Pearson’s product-moment correlation coefficients provided a measure of the co-linear relationship between the oxyhemoglobin (HbO) signals of different channels over the PFC that have been measured on interval scales. A lower “all-channels” index of functional connectivity (decreased mean *r*-values) provides evidence of desynchronization, indicating that hemodynamic changes occurred at unrelated times resulting in higher frequency of brain oscillations. The higher-frequency oscillations (desynchrony) have been linked to increased hemodynamic activity in cat, monkey, and human studies (Kahana, 2006). In contrast, a higher functional connectivity (increased mean *r*-values) provides evidence of synchronization, which means the relation that exists when signals occur at corelated times, resulting in lower frequency of brain oscillations. At the functional level, attention and working memory tasks desynchronize brain oscillations, whereas quiet rest promotes synchrony of brain oscillations (Kahana, 2006). Similarly, our recent study (Barrett et al., 2025) found that the “all-channels” index of functional connectivity was significantly lower during the 2-back memory phase than during the rest phase of the fNIRS procedure in adults with remitted bipolar disorder.

Notably, significant effects of TILS on functional connectivity were only found during the 2-back cognitive activation task, and not during the rest phase. This may be due to the increased metabolic demand resulting from cognition during the task, with TILS providing additional energy to meet this increased demand via photo-oxidation of cytochrome-c-oxidase (CCO) and increased hemodynamic HbO concentrations (Wang et al., 2017). This finding may also be due to TILS accelerating the changes in functional connectivity that underlie the learning process, which would not be found during the rest phase. Learning results in neuroplastic changes in functional brain networks which are not found during the resting state (Klug et al., 2022), with increasing training resulting in a relatively sparse representation (Grill-Spector et al., 2006) as the brain becomes more efficient in the particular cognitive process. TILS may be facilitating this shift, which is reflected in decreased functional connectivity in prefrontal cortical region.

Transcranial infrared laser stimulation resulted in a pattern of neuroplastic changes in PFC-based memory functional connectivity that was different than that found in the sham-treated group. The TILS-treated group showed relative decreases in PFC functional connectivity, while the sham-treated group developed a higher functional connectivity across days. This shift toward more desynchronized processing because of TILS is evident during the first and second order sequence of the experiment. Even after the washout period, and after the activation task was well-learned, TILS resulted in a lower functional connectivity relative to the sham-treated group. This finding is consistent with BOLD-fMRI research showing that engaging in the 2-back memory task tends to cause decreases in average whole-brain measures of functional connectivity as compared to the resting state (Stanley et al., 2015). Thus, TILS may facilitate this shift from synchrony to desynchrony from the increased cognitive demand during the task phase.

Since these effects were unique to the memory activation phase, they may represent a shift between brain modes of cognitive processing, with higher functional connectivity indicating more synchronization in PFC processing, and lower functional connectivity indicating more desynchronized PFC processing. TILS may be facilitating this process, with an increase in local desynchrony indicating greater metabolic capacity for the PFC’s mediation of cognitive task processing.

Transcranial infrared laser stimulation-enhanced desynchronization of fNIRS activity in the PFC may be interpreted as a signature of enhanced metabolic capacity because desynchronization typically indexes a shift toward information-rich, energetically demanding processing. In many cortical regions, desynchrony reflects a transition from coordinated, rhythmic firing to more independent and diverse neuronal activity, allowing neurons to encode a larger variety of inputs and increase representational flexibility. This interpretation aligns with the “information via desynchronization” framework, which posits that desynchronized population activity supports greater information throughput at the cost of higher energetic demand (Hanslmayr et al., 2012). Neural signaling is metabolically expensive, primarily because restoring ion gradients after action potentials and synaptic transmission consumes substantial ATP. Consequently, states characterized by increased firing variability, higher synaptic throughput, and reduced population synchrony typically require greater metabolic support (Laughlin et al., 1998).

Computational and theoretical work further strengthens the connection between desynchronization and metabolic capacity. Joo et al. (2021) demonstrated that when ATP production is experimentally reduced in neural network models, the system transitions into more synchronized firing patterns. Conversely, higher ATP availability allows neurons to maintain desynchronized firing regimes. This suggests that desynchronization is not metabolically neutral but instead reflects a well-supported energetic environment. Similar principles emerge in modeling studies showing that energetic constraints shape network connectivity and dynamic range, such that networks with greater available metabolic resources can sustain richer, more flexible activity patterns (Takagi, 2021). Recent theoretical work further links enhanced metabolic capacity to the stability of attractor landscapes in cortical circuits, implying that desynchronized states, common in the PFC during cognitive tasks, depend on adequate energy supply (Buxton and Wong, 2024).

Empirical work in the PFC supports these theoretical interpretations. The PFC is among the most metabolically demanding brain regions due to its dense connectivity, high synaptic activity, and role in complex cognition. Studies demonstrate that metabolic demands in the PFC increase under cognitive load or stress, with associated increases in glucose utilization and enzymatic activity supporting synaptic function (Musazzi et al., 2019). Given that desynchronized states generally reflect increased synaptic and spiking activity, observing local desynchronization in PFC circuits is consistent with enhanced metabolic support enabling complex, high-capacity information processing. Together, these findings suggest that local desynchronization may be a functional indicator of underlying metabolic capacity, with energetic sufficiency enabling the flexible, dynamic neural computations characteristic of the PFC.

### Limitations

We acknowledge the limitations of using a global average. However, to evaluate potential hemispheric regional differences that may exist due to functional specialization, the same repeated measures ANCOVA was repeated with an additional within-subject variable: right vs. left hemisphere. This hemispheric analysis did not reveal any significant interactions with the right/left variable. In addition, the effects in each hemisphere showed similar trends to those observed in the global average. Notably, the sample size is limited, which constrains generalizability.

A 4-week washout period may have been insufficient in eliminating carry-over effects in activation task performance and functional connectivity. Our sample size was insufficient to evaluate the acute effect of TILS on functional connectivity; this question was outside the scope of this study and should be better addressed in future work. The 2-back task, while effective as a source of cognitive activation and increased metabolic demand, was too easy to master and thus resulted in ceiling effects in performance. Future studies evaluating TILS as a neurocognitive enhancer may benefit by including a more difficult activation task, to drive greater metabolic energy demand and better serve as a dependent variable to evaluate the effects of TILS on cognitive performance and functional connectivity.

## Conclusion

The present study provided the first evidence for a prolonged neuromodulatory effect of a single administration of TILS on memory activated functional connectivity in the PFC over a 5-day period. It also helped to illustrate fNIRS-derived functional connectivity as a promising *in vivo* mechanism by which to non-invasively assess duration of action of PBM across repeated measurements. This could guide future research into TILS longer duration of action and optimal dosing for clinical applications.

## Data Availability

The raw data supporting the conclusions of this article will be made available by the authors, without undue reservation.
